# Cricothyroidotomy in out-of-hospital cardiac arrest: An observational study

**DOI:** 10.1016/j.resplu.2024.100833

**Published:** 2024-11-26

**Authors:** Matthew Humar, Benjamin Meadley, Bart Cresswell, Emily Nehme, Christopher Groombridge, David Anderson, Ziad Nehme

**Affiliations:** a Ambulance Victoria, 375 Manningham Rd, Doncaster, Melbourne, Victoria 3108, Australia; b Department of Paramedicine, Monash University, Level 2, Building H, Peninsula Campus, 47-49 Moorooduc Hwy, Frankston, Victoria 3199, Australia; c School of Public Health and Preventive Medicine, Monash University, 553 St Kilda Rd Melbourne, Victoria 3004, Australia; d School of Translational Medicine, Monash University, Level 6, Alfred Centre, 99 Commercial Rd, Melbourne, Victoria 3004, Australia; e National Trauma Research Institute, Level 4/89 Commercial Rd, Melbourne, Victoria 3004, Australia; f The Alfred Hospital, Alfred Health, 55 Commercial Rd, Melbourne, Victoria 3004, Australia

**Keywords:** Emergency medical services, Out-of-hospital cardiac arrest, Cardiopulmonary resuscitation, Emergency front-of-neck access, Difficult airway, Cricothyroidotomy

## Abstract

**Aim:**

To describe the incidence, characteristics, success rates, and outcomes of out-of-hospital cardiac arrest (OHCA) patients receiving cricothyroidotomy.

**Methods:**

Over an 18-year period, we retrospectively analysed patient care records and cardiac arrest registry data for cricothyroidotomy cases. Multivariable logistic regression analysis was used to examine associations between study characteristics and cricothyroidotomy success.

**Results:**

We identified 80 cricothyroidotomies, 56 of which occurred in OHCA. The incidence of cricothyroidotomy in OHCA was 1.1 per 1,000 attempted resuscitations and increased over the study period (incidence rate ratio [IRR] = 1.13, 95 % confidence interval [CI]: 1.02–1.25, p = 0.023). The overall success rate was 68.8 % (n = 55/80), with lower success in cardiac arrest (n = 33/56, 58.9 %) than non-cardiac arrest patients (n = 22/24, 91.7 %). In OHCA, success rates were higher for surgical compared to needle techniques (88.2 % vs. 54.6 %, p = 0.003). Cardiac arrest (odds ratio [OR] 0.09, 95 % CI 0.16–0.51) and needle techniques (OR 0.11, 95 % CI 0.02–0.56) were independently associated with lower odds of procedural success, while male sex (OR 10.06, 95 % CI 2.00–50.62) was associated with higher odds. Return of spontaneous circulation occurred in 44.6 % (n = 22/56), with 35.7 % (n = 20/56) surviving to hospital and 7.1 % (n = 4/56) surviving to hospital discharge. Procedural complications included cardiac arrest (n = 6/56, 10.7 %), minor bleeding (n = 5/56, 8.9 %), surgical emphysema (n = 3/56, 5.4 %), and major bleeding (n = 2/56, 3.6 %).

**Conclusion:**

We found cricothyroidotomy in OHCA to be associated with low rates of procedural success and high mortality rates. Further studies are required to assess the role and potential benefits of cricothyroidotomy in cardiac arrest.

## Introduction

Cricothyroidotomy, an emergency front-of-neck access (eFONA) technique, is a life-saving intervention where a breathing tube is inserted through the cricothyroid membrane into the trachea to facilitate oxygen delivery.^1^ Indicated for ‘can’t intubate, can’t oxygenate’ situations,^2^ cricothyroidotomy is also a primary airway technique when face-mask ventilation, supraglottic airway (SGA) placement, and tracheal intubation are impossible. In both scenarios, it is the last resort to restore oxygenation and prevent hypoxic injury.^3^

Systematic reviews and meta-analyses indicate that surgical eFONA techniques are more successful than needle techniques in the adult prehospital population.^4^, ^5^ Due to high success and low complication rates, along with other procedural benefits, surgical techniques are also widely recommended by anaesthesia guidelines.^6^, ^7^ For paediatric patients, there is currently limited evidence to support an optimal prehospital eFONA technique.^5^, ^8^ Similarly, evidence on eFONA in out-of-hospital cardiac arrest (OHCA) is lacking with a recent scoping review by the International Liaison Committee on Resuscitation (ILCOR) Advanced Life Support (ALS) Task Force finding no studies examining the role of cricothyroidotomy in cardiac arrest.^9^

Using non-cardiac arrest cases as a control, this study aimed to describe the incidence, success, timing, outcomes, and complications of cricothyroidotomy in OHCA.

## Methods

### Study design

We conducted a retrospective observational cohort study of patients that underwent cricothyroidotomy between 7 November 2005 and 30 June 2024. Patients of all ages were included in our analysis. We excluded cases involving intubation through a tracheal laceration/puncture or tracheostomy stoma, and where cricothyroidotomy occurred prior to paramedic arrival. Cricothyroidotomy by non-paramedics (e.g., physicians) were also excluded. Physicians in our EMS do not respond to primary emergency road or air incidents, but instead provide retrieval services. Thus, cases of physician-performed cricothyroidotomy were not representative of the sample under investigation.

This study was approved by the Ambulance Victoria Research Committee and Monash University Human Research Ethics Committee (Victoria, Australia; project ID 41909), and is reported in accordance with the Strengthening the Reporting of Observational Studies in Epidemiology (STROBE) guidelines.^10^

### Setting

Ambulance Victoria is the sole emergency medical service (EMS) provider for the state of Victoria, Australia, servicing over 6.6 million people across an area of 227,500 km^2^.^11^ Predominantly staffed by advanced life support (4,397 full-time equivalents) and intensive care paramedics (463 full-time equivalents), in 2023, Ambulance Victoria responded to over 680,000 emergency road incidents and 7,800 OHCA events.^12^, ^13^ Additionally, Air Ambulance Victoria, a subsidiary of Ambulance Victoria, dispatches fixed-winged aircraft or helicopters to approximately 7,800 incidents per year.

Ambulance Victoria intensive care paramedics (ICPs) are ground-based prehospital clinicians who can perform advanced airway management interventions and procedures. These include i-gel™ supraglottic airway insertion (Intersurgical Ltd, Wokingham, Berkshire, UK), unassisted tracheal intubation, sedation-facilitated intubation (for patients < 12 years of age), and rapid sequence intubation (for patients ≥ 12 years). ICPs can also perform surgical cricothyroidotomy for patients older than 12 years.

Intensive care flight paramedics (ICFPs), who staff aeromedical resources (rotary and fixed wing), have an expanded critical care clinical skill set.^14^ In addition to the above, this includes use of intubating laryngeal mask airways (LMA-Fastrach; Intavent Ltd, Reading, UK) and rapid sequence intubation for all ages. They can also perform cannula cricothyroidotomy for paediatric patients and provide jet ventilation using the Rapid-O2™ insufflation oxygen delivery device (Meditech Systems Ltd, Dorset, UK). Both ICPs and ICFPs use continuous waveform capnography to confirm tracheal tube position, and practice according to service-specific clinical practice guidelines.^15^

Throughout the study period, advanced airway management equipment and service-guidelines changed on several occasions. These changes included introduction of the i-gel™ supraglottic airway device (2012) and McGrath™ MAC® video laryngoscope (2015; Medtronic, Dublin, Ireland), as well as updates to medication-facilitated intubation (2020) and difficult airway guidelines (2023). Cricothyroidotomy technique also evolved during this time. Specifically, in 2015, the Rusch® QuickTrach II™ (VBM, Medizintechnik GmbH®, Sulz am Neckar, Germany) superseded the Portex Mini-Trach® kit (Smith’s Medical; Smiths Medical Ltd, Hythe, UK), and, in 2019, cannula cricothyroidotomy was added to ICFP practice. Furthermore, in 2021 and 2022, the scalpel-finger-bougie method for cricothyroidotomy was introduced to ICFP and ICP practice, respectively.^16^ In our EMS, a first-pass intubation success rate of 84 % and an overall success rate of 95 % have been reported in cardiac arrest cases.^13^, ^17^

### Data sources

To locate relevant cases, we searched and extracted all electronic patient care records (PCRs) that involved cricothyroidotomy. Electronic data capture did not exist in this EMS prior to 7 November 2005; thus no PCRs are included in this study before this date.

As described elsewhere,^18^ paramedics complete a PCR following each patient encounter, with all records uploaded and stored in the Ambulance Victoria Clinical Data Warehouse. Occasionally, paramedics complete written PCRs in lieu of electronic records (e.g., due to computer malfunction). We did not search written records for cricothyroidotomy cases. Once relevant cases were identified, we matched these with records from the Victorian Ambulance Cardiac Arrest Registry (VACAR). The VACAR includes Utstein-style descriptors for all OHCA patients attended by EMS in Victoria. Moreover, as part of standard data linkage processes, VACAR routinely receives discharge diagnoses and outcomes for all patients transported to hospital. This registry has been described previously.^19^

From the above sources, we extracted electronic data elements on location, patient demographics, operator skill set, and timing intervals. Additionally, we reviewed PCRs to manually extract information on advanced airway interventions prior to cricothyroidotomy and the cricothyroidotomy procedure. For this, two abstractors used a standardised data extraction form created in Microsoft Excel V.16.83 (Microsoft, Washington, USA), which was then merged with VACAR registry data.

### Outcomes and definitions

A cricothyroidotomy attempt was defined as the insertion of a needle, proprietary cricothyroidotomy device, or tracheal tube through the anterior neck, with or without a surgical incision. We further classified needle techniques as those using either a narrow- or wide-bore cannula, and surgical techniques as those involving the scalpel-finger-bougie method. In cases where technique data was missing (n = 7), we applied the approved cricothyroidotomy technique in-practice at that time.

Procedural success was defined as adequate ventilation following placement as determined by continuous waveform capnography. In some instances, this resulted in patients with complete tracheal obstruction being classified as unsuccessful. Although available for some patients, we did not access online recordings of waveform capnography to confirm cricothyroidotomy success. Success rates are calculated per individual patient, not per procedure.

Like previous airway studies, primary cricothyroidotomy was defined as cricothyroidotomy without preceding attempts at tracheal intubation.^20^, ^21^ Conversely, we defined rescue cricothyroidotomy as unsuccessful tracheal intubation attempts followed by cricothyroidotomy. Outcomes and complications were informed by Utstein-style reporting templates for both cardiac arrest and airway management.^22^, ^23^

Time to cricothyroidotomy was defined as the interval between EMS arrival on scene and the first cricothyroidotomy attempt.

### Statistical analyses

Statistical analyses were undertaken using Stata Statistical Software v.18 (StataCorp LLC, College Station, TX, 2023). All p-values were two-tailed, and a p-value < 0.05 was considered statistically significant. To calculate cricothyroidotomy incidence rates, we used two different denominators: 1) patient encounters that resulted in an electronic PCR, and 2) attempted cardiac arrest resuscitations. Except for 2006, incidence rates were calculated biennially (i.e., two consecutive fiscal years). This aimed to smooth annual fluctuations of this rare event and to better capture change over time.

Descriptive statistics were used to summarise findings. Numerical data were reported as either mean and standard deviation or median and interquartile range, as appropriate. Categorical data are presented as frequencies and proportions. For categorial variables, group differences were compared using either the chi-square (χ2) or Fisher’s exact test, while for continuous variables we used an unpaired *t*-test or Mann-Whitney *U* test. A Poisson regression was used to examine trends in the incidence and success rates of cricothyroidotomy, with incidence rate ratios (IRRs) and 95 % confidence intervals reported.

Additionally, we developed a multivariable logistic regression model to examine the relationship between study characteristics and cricothyroidotomy success. Patient and procedural characteristics that were significantly associated with cricothyroidotomy success in univariable analysis were included in the multivariable model. Results from these models were reported as odds ratios and 95 % confidence intervals.

## Results

### Sample population

**Fig. 1:**
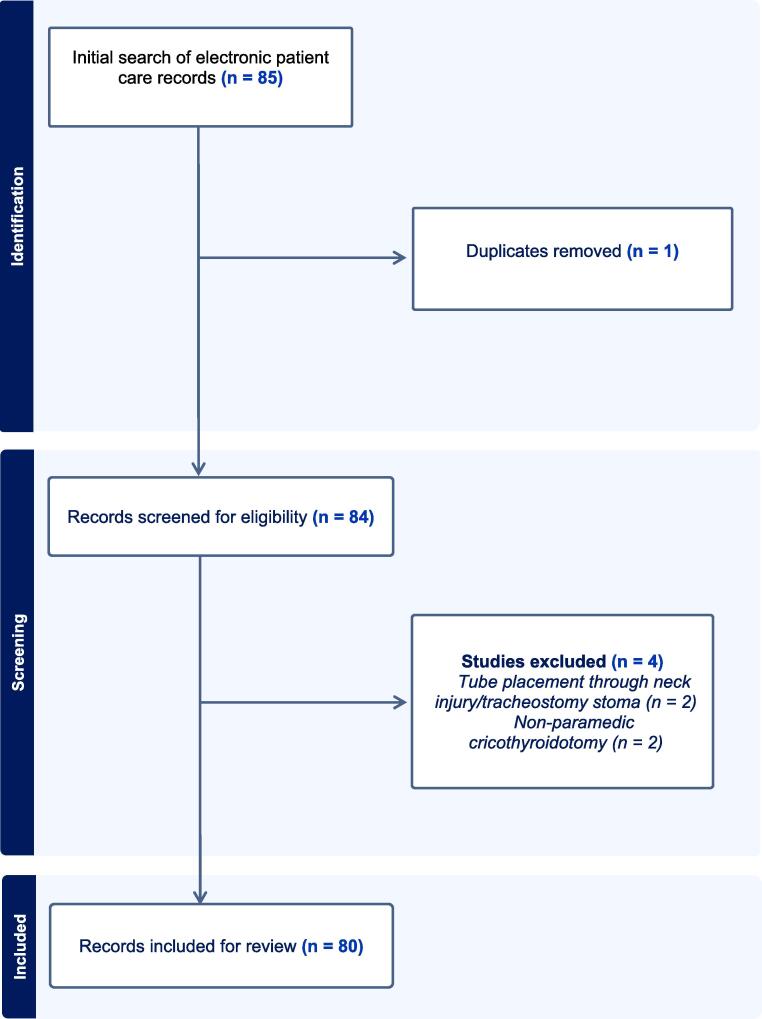
Study population flow-chart.

We identified 85 cases of cricothyroidotomy. Five of these cases were excluded because they were duplicates (n = 1), involved non-paramedic cricothyroidotomy (n = 2), or involved tube placement through a pre-existing neck injury or tracheostomy stoma (n = 2). This resulted in 80 patients being included in the study for analysis (Fig. 1).

### Incidence

During the study period, a total of 8,796,761 patient encounters resulted in the generation of electronic PCRs. Among these, 80 involved cricothyroidotomy: 56 cardiac arrest patients and 24 non-cardiac arrest patients. Accordingly, the overall incidence of cricothyroidotomy in this study was 0.01 per 1,000 patient encounters. Fig. 2a shows the frequency of cricothyroidotomy and provides a breakdown of its frequency and success in OHCA patients.

**Fig. 2a:**
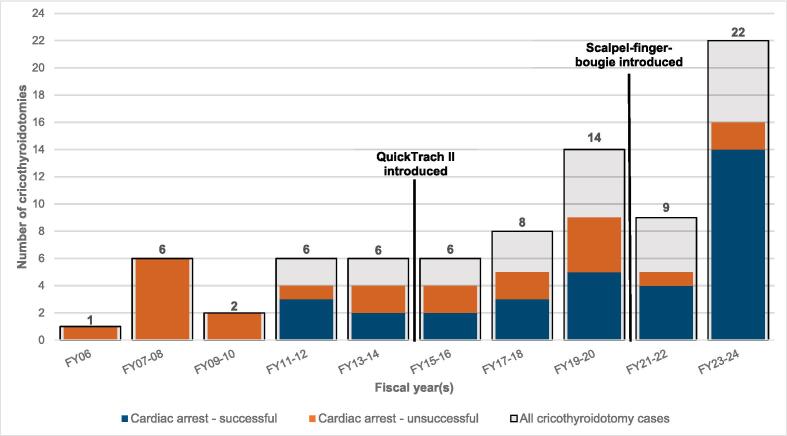
Incidence and success of cricothyroidotomy. Numbers and grey boxes indicate all cases, while coloured boxes indicate cardiac arrest cases and success.

During the study period, there were 50,981 OHCA resuscitation attempts. Fig. 2b shows the incidence of cricothyroidotomy per 1,000 attempted resuscitations. As shown, the incidence of cricothyroidotomy in OHCA increased over time, peaking at 2.53 per 1,000 attempted resuscitations in FY2023-24. This represented a 13.0 % increase per biennial interval (IRR = 1.13; 95 % CI: 1.02–1.25, p = 0.023).

**Fig. 2b:**
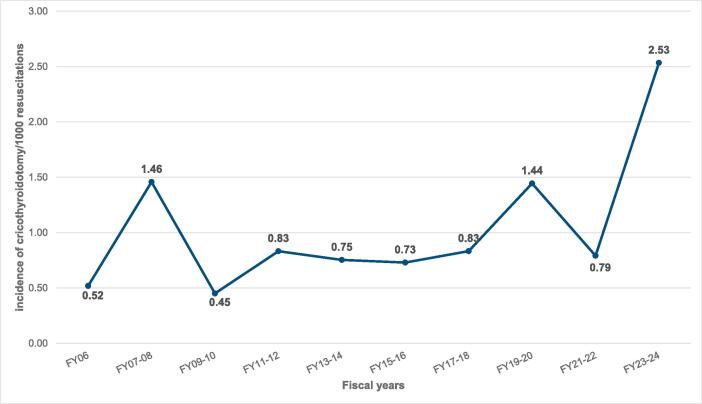
Incidence of cricothyroidotomy in cardiac arrest per 1,000 resuscitations (N = 50,981).

### Patient characteristics

Patient characteristics are summarised in Table 1. The cardiac arrest group predominately consisted of males (n = 45/56, 80.4 %) with a mean age of 51.2 (standard deviation [SD] 20.8) years. Medical aetiology was more common in cardiac arrest compared to non-cardiac arrest (69.6 % vs. 8.3 %, p < 0.001), whilst trauma was more prevalent in the non-cardiac arrest group (30.4 vs. 91.7 %, p < 0.001). Significant between-group differences were also seen in some medical and trauma sub-categories, including foreign body airway obstruction (p = 0.028), transportation accident (p = 0.027), burns injury (p = 0.008), and other causes (p = 0.027).

**Table 1:** Patient and paramedic characteristics.

Characteristic	Cardiac arrest N = 56	Non-cardiac arrest N = 24	Total N = 80	p-value	Missing, n (%)
Demographic					
Age (years), mean (SD)	51.2 (20.8)	37.6 (13.8)	47.2 (20.0)	**0.006**	2 (2.5)
Adult ≥ 16 years, n (%)	52 (94.6)	22 (95.7)	74 (94.9)	0.840	2 (2.5)
Male sex, n (%)	45 (80.4)	20 (83.3)	65 (81.3)	0.755	−
Weight (kg), median (IQR)	90 (70–110)	80 (70–100)	80 (70–100)	0.475	18 (23.1)
Urban region, n (%)	38 (67.9)	12 (50.0)	50 (62. 5)	0.131	−
Paramedic skill set					
ICP, n (%)	51 (91.1)	13 (54.2)	64 (80.0)	**<0.001**	−
ICFP, n (%)	5 (8.9)	11 (45.8)	16 (20.0)	**<0.001**	−
Patient category					
Medical, n (%)	39 (69.6)	2 (8.3)	41 (51.3)	**<0.001**	−
Upper airway swelling^a^	12 (21.4)	1 (4.2)	13 (16.3)	0.095	−
FBAO	11 (19.6)		11 (13.8)	**0.028**	−
Cardiovascular event	10 (17.9)		10 (12.5)	**0.028**	−
Other^b^	6 (10.7)	1 (4.2)	7 (8.8)	0.668	−
Trauma, n (%)	17 (30.4)	22 (91.7)	39 (48.8)	**<0.001**	−
Transportation accident	12 (21.4)	11 (45.8)	23 (28.8)	**0.027**	−
Burns injury	2 (3.6)	6 (25.0)	8 (10.0)	**0.008**	−
Penetrating injury	2 (3.6)	1 (4.2)	3 (3.8)	1.000	−
Other^c^	1 (1.8)	4 (16.7)	5 (6.3)	**0.027**	−
Cricothyroidotomy indication, n (%)					
Soiled airway	11 (19.6)	−	11 (13.8)	**0.028**	−
Major facial/neck trauma	10 (17.9)	12 (50.0)	22 (27.5)	**0.003**	−
Obstructed airway (food or object)	10 (17.5)		10 (12.5)	**0.028**	−
Upper airway distortion^d^	10 (17.9)	7 (29.2)	17 (21.3)	0.371	−
Other^e^	15 (26.8)	5 (20.8)	20 (25.0)	0.573	−

Key: SD – standard deviation, IQR – interquartile range, ICP – intensive care paramedic, ICFP – intensive care flight paramedic, FBAO – foreign body airway obstruction.

a Includes abnormal airway anatomy, expanding neck hematoma, and airway swelling secondary to anaphylaxis, infection, or unknown cause.

b Includes acute poisoning, respiratory disease, and seizure disorders.

c Includes falls, struck by object, and asphyxiation.

d Includes burn injury, abnormal airway anatomy, expanding neck hematoma, and airway swelling secondary to anaphylaxis, infection, or unknown cause.

e Includes non-pathological anatomical difficult airways, trismus/strong jaw tone, and miscellaneous can’t intubate, can’t oxygenate (CICO) situations.

A soiled (n = 11/56, 19.6 %) or obstructed airway (n = 10/56, 17.5 %) that could not be cleared were common indications for cricothyroidotomy in cardiac arrest patients. Conversely, non-cardiac arrest patients had high rates of cricothyroidotomy secondary to major facial/neck trauma (n = 12/24, 50.0 %).

### Cardiac arrest characteristics

Table 2 details cardiac arrest characteristics. Cardiac arrest occurred before and after cricothyroidotomy in 89.3 % (n = 50/56) and 10.7 % (n = 6/56) of cases, respectively. The majority of OHCA also occurred at a private residence (n = 32/56, 57.1 %) and were bystander witnessed (n = 29/56, 51.8 %), with bystander CPR provided in 46.4 % (n = 26/56) of cases. A presumed cardiac aetiology for OHCA was identified in 17.9 % (n = 10/56) of cases, while 87.3 % (n = 48/56) of patients presented in a non-shockable initial arrest rhythm. The median (IQR) EMS response time was 8.1 (6.8–12.5) minutes.

**Table 2:** Cardiac arrest characteristics and survival outcomes.

Characteristic	Cardiac arrest, n (%) (N = 56)	Missing, n (%)
Timing of cardiac arrest, n (%)		−
Before cricothyroidotomy	50 (89.3)	
After cricothyroidotomy	6 (10.7)	
Arrest location, n (%)		
Public	16 (28.6)	−
Private residence	32 (57.1)	−
Aged care	2 (3.6)	−
Other	6 (10.7)	−
Presumed cardiac etiology, n (%)	10 (17.9)	−
Witnessed, n (%)		
EMS	22 (39.3)	−
Bystander	29 (51.8)	−
Not witnessed	5 (8.9)	−
Bystander CPR, n (%)	26 (46.4)	−
Initial/arrest rhythm, n (%)		1 (1.8)
Shockable	7 (12.7)	−
Non-shockable	48 (87.3)	−
EMS response time (min), median (IQR)	8.1 (6.8–12.5)	−
Resuscitation duration (min), median (IQR)	29.0 (17.0–39.0)	3 (5.4)
Scene outcomes		
Died at scene	33 (58.9)	−
Transported with CPR	3 (5.4)	−
Transported with ROSC	20 (35.7)	
Survival outcomes, n (%)		
Pre-hospital ROSC	25 (44.6)	−
Survival to hospital	20 (35.7)	−
Discharged alive	4 (7.1)	−
Direction		
Home	3 (5.4)	−
Rehabilitation	1 (1.8)	−

Key: EMS – emergency medical service, CPR – cardiopulmonary resuscitation, IQR – interquartile range, ROSC – return of spontaneous circulation

Of the 56 cardiac arrest patients, 44.6 % (n = 25/56) achieved return of spontaneous circulation (ROSC), 35.7 % (n = 20/56) survived to hospital, and 7.1 % (n = 4/56) were discharged from hospital alive. Among patients whose cricothyroidotomy occurred during cardiac arrest and before ROSC (n = 47/56, 83.9 %), 40.4 % (n = 19/47), achieved ROSC, 31.9 % (n = 15/47) survived to hospital, and 6.4 % (n = 3/47) survived to hospital discharge.

### Procedural characteristics

Procedural characteristics are summarised in Table 3. Rapid sequence intubation preceded cricothyroidotomy in 66.7 % (n = 16/24) of non-cardiac arrest cases compared to 14.3 % (n = 8/56) of cardiac arrest cases (p < 0.001). In OHCA cases, RSI was either performed before cardiac arrest (n = 5) or during ROSC (n = 3). A higher proportion of cardiac arrest patients had more than two intubation attempts prior to cricothyroidotomy (34.6 % vs. 4.2 %, p = 0.004). There were no significant between-group differences in video laryngoscopy or SGA use, primary or rescue classification, or cricothyroidotomy technique.

**Table 3:** Procedural characteristics.

Characteristic	Cardiac arrest N = 56	Non-cardiac arrest N = 24	Total N = 80	p-value	Missing, n (%)
Airway intervention prior to cricothyroidotomy					
RSI, n (%)	8 (14.3)	16 (66.7)	24 (30.0)	**<0.001**	−
Number of intubation attempts, n (%)					1 (1.3)
0	13 (23.6)	8 (33.3)	21 (26.6)	0.346	
1	11 (20.0)	4 (16.7)	15 (19.0)	0.755	
2	12 (21.8)	11 (45.8)	23 (29.1)	**0.027**	
≥ 3	19 (34.6)	1 (4.2)	20 (25.3)	**0.004**	
Video laryngoscope used, n (%)	21 (37.5)	9 (37.5)	30 (37.5)	1.000	
SGA attempted prior to cricothyroidotomy, n (%)	27 (48.2)	11 (45.8)	38 (47.5)	0.845	
Cricothyroidotomy					
Primary or rescue, n (%)					
Primary	13 (23.2)	8 (33.3)	21 (26.3)	0.346	
Rescue	43 (76.8)	16 (66.7)	59 (73.8)	0.346	
Number of cricothyroidotomy attempts, n (%)					1 (1.3)
1	39 (70.9)	21 (87.5)	60 (76.0)	0.091	
2	12 (21.8)	2 (8.3)	14 (17.7)	0.158	
≥ 3	4 (7.3)	1 (4.2)	5 (6.3)	1.000	
Technique^a^, n (%)					
Needle	39 (69.6)	17 (70.8)	56 (70.0)	0.915	
Surgical	17 (30.4)	7 (29.2)	24 (30.0)	0.915	
Time to cricothyroidotomy (min), median (IQR)	21 (14.5–34.5)	35 (22–75.5)	24.5 (16.5–41.5)	**<0.001**	
Success, n (%)					
First attempt success	27 (48.2)	19 (79.2)	46 (57.5)	**0.010**	
Overall success	33 (58.9)	22 (91.7)	55 (68.8)	**0.004**	

Key: RSI – rapid sequence intubation, SGA – supraglottic airway, IQR – interquartile range.

a Frequencies and proportions based on initial cricothyroidotomy attempt.

### Success rates

During the study period, cricothyroidotomy success rates increased by 21.0 % per biennial interval (IRR = 1.21; 95 % CI: 1.04–1.40, p = 0.015). Our overall cricothyroidotomy success rate was 68.8 % (n = 55/80), with significantly lower success in cardiac arrest compared to the non-cardiac patients (58.9 % vs. 91.7 %, p = 0.004) (Table 3). Additionally, surgical techniques were more successful than needle techniques on first attempt (82.4 % vs. 33.3 % vs. p = 0.001) and overall (88.2 % vs. 46.2 %, p = 0.003). Cricothyroidotomy was classified as unsuccessful in 14.2 % (n = 8/56) cases of complete tracheal obstruction.

Table 4 shows the univariable and multivariable analysis for characteristics associated with cricothyroidotomy success. Characteristics independently associated with the odds of cricothyroidotomy success included male sex (OR, 10.06; 95 % CI, 2.00–50.62), needle techniques (OR, 0.11; 95 % CI, 0.02–0.56), and cardiac arrest (OR, 0.09; 95 % CI, 0.16–0.51).

**Table 4:** Univariable and multivariable analyses for cricothyroidotomy success (N = 80).

Characteristic	Univariable analysis	p-value	Multivariable analysis	p-value
	**OR (95 %CI)**		**OR (95 %CI)**	
Age	0.98 (0.96–1.01)	0.130	−	−
Weight	1.00 (0.98–1.01)	0.671	−	−
OHCA				
Non-cardiac arrest	Reference			
Cardiac arrest	0.13 (0.03–0.61)	**0.010**	0.09 (0.16–0.51)	**0.007**
Sex				
Female	Reference			
Male	4.59 (1.42–14.91)	**0.011**	10.06 (2.00–50.62)	**0.005**
Region				
Rural	Reference			
Urban	0.54 (0.19–1.51)	0.240	−	−
Skill set				
ICFP	Reference			
ICP	0.44 (0.11–1.71)	0.237	−	−
Patient category				
Trauma	Reference			
Medical	0.47 (0.18–1.24)	0.127	−	−
Primary or rescue				
Primary	Reference			
Rescue	0.61 (0.19–1.90)	0.394	−	−
Technique				
Surgical	Reference			
Needle	0.22 (0.06–0.83)	**0.025**	0.11 (0.02–0.56)	**0.008**

Key: OR – odds ratio, 95%CI – 95% confidence interval, ICFP – intensive care flight paramedic, ICP – intensive care paramedic.

### Complications

Thirteen out of fifty-six (23.2 %) OHCA patients experienced a procedure-related complication. Documented complications included cardiac arrest (n = 6/56, 10.7 %; for those with spontaneous cardiac output at the time of the procedure), minor bleeding (n = 5/56, 8.9 %), subcutaneous emphysema (n = 3/56, 5.4 %), and major bleeding (n = 2/56, 3.6 %). By technique, cardiac arrest occurred in five out of six cases of needle cricothyroidotomy. Minor bleeding was observed in four out of five cases of surgical cricothyroidotomy, while major bleeding occurred in one case of surgical cricothyroidotomy and another involving both techniques. All instances of subcutaneous emphysema were associated with needle techniques.

## Discussion

Focusing on OHCA, this study sought to describe the role of cricothyroidotomy in patients managed by a statewide Australian emergency medical service (EMS). In doing so, we aimed to address an identified ILCOR knowledge gap and provide initial insights into this rarely performed yet critical intervention.

The overall incidence of cricothyroidotomy in this study was 0.01 per 1,000 patient encounters or 1.1 per 1,000 attempted OHCA resuscitations. Furthermore, the highest incidence of cricothyroidotomy in OHCA that was recorded during the 18-year study period occurred in 2023–24, reaching 2.53 per 1,000 attempted resuscitations. This is the first report to characterise cricothyroidotomy incidence per attempted resuscitations, limiting direct comparisons with similar studies. Nonetheless, this trend represents a significant change from previous years and contrasts with other reports where improvements in training and equipment have been associated with reduced cricothyroidotomy incidence rates.^21^, ^24^, ^25^

Although the reasons for this finding are unclear, we propose three potential explanations. First, an increase in cases involving difficult airway management might have heightened the clinical need for cricothyroidotomy during this period. Second, between 2022 and 2024, there were service-wide changes to cricothyroidotomy training and procedure, including guideline updates and the introduction of a surgical technique for ICPs. These changes, supported by training and mandatory annual credentialing, may have increased paramedic confidence and willingness to perform this intervention.^26^ Lastly, as alluded in similar reports,^27^, ^28^ the introduction of a new cricothyroidotomy technique may have resulted in ‘the technical imperative’—the performance of an intervention more frequently than indicated.^29^ However, since we did not analyse the appropriateness of cricothyroidotomy, this explanation remains speculative.

Our overall cricothyroidotomy success rate was 68.8 %, with significantly lower success in cardiac arrest compared to non-cardiac arrest patients (58.9 % vs. 91.7 %). While this rate (68.8 %) is relatively low in comparison to the pooled success rate of 88.0 % reported by Morton et al.,^5^ the number of cardiac arrest patients included in this systematic review is unknown. Consistent with other reviews, we found that needle techniques had lower success rates than surgical techniques (46.2 % vs 88.2 %).^4^, ^5^ Correspondingly, our multivariable analysis model showed that the odds of successful cricothyroidotomy were 91.0 % (OR, 0.09; 95 % CI, 0.16–0.51) lower for cardiac arrest compared to non-cardiac arrest patients, and 89.0 % (OR, 0.11; 95 % CI, 0.02–0.56) lower for needle compared to surgical techniques. This model also showed the odds of successful cricothyroidotomy were 10 times higher in males than females (OR, 10.06; 95 % CI, 2.00–50.62). These findings may reflect the technical challenges of performing cricothyroidotomy, especially with needle techniques, during OHCA resuscitation (e.g., sub-optimal positioning, chest compression interference), and the difficulty of correctly identifying the cricothyroid membrane in female patients.^30^, ^31^, ^32^

In comparison to OHCA clinical outcomes in Australia and New Zealand, our sample had high rates of ROSC (44.6 %) and survival to hospital admission (35.7 %).^33^ Favourable arrest characteristics, such as EMS- or bystander-witnessed cardiac arrest (91.1 %), bystander CPR (46.4 %), and a median EMS response time of 8.1 min potentially influenced these findings.^34^, ^35^, ^36^ Despite these positive initial outcomes, only four patients (7.1 %) survived to hospital discharge. Consistent with other prehospital reports, no patients in traumatic cardiac arrest who underwent cricothyroidotomy survived.^28^, ^37^, ^38^ This likely reflects the poor prognosis following traumatic cardiac arrest, particularly from transportation accidents, which were common in our sample.^39^ Our results, in general, challenge the value of cricothyroidotomy in patients that suffer cardiac arrest in trauma.^38^, ^40^

The low survival rate in our study may have been influenced by several factors. These include the predominance of non-shockable rhythms (87.3 %), a low incidence of cardiac causes (17.9 %), and the high percentage of patients who were in cardiac arrest at the time of cricothyroidotomy (83.9 %). Together, these factors characterise a cohort of critically ill patients with inherently low survival rates.^38^, ^41^, ^42^ Another possible contributing factor is the time required to perform cricothyroidotomy in OHCA patients. The median response time and time from EMS arrival to cricothyroidotomy were 8.1 and 21.0 min, respectively. Inadequate ventilation during this time could lead to prolonged hypoxia and negative patient outcomes.^43^, ^44^, ^45^

Complications of cricothyroidotomy were identified in 23.2 % (n = 13/56) of OHCA cases, and included cardiac arrest (n = 6/56, 10.7 %), minor bleeding (n = 5/56, 8.9 %), subcutaneous emphysema (n = 3/56, 5.4 %), and major bleeding (n = 2/56, 3.6 %). Notably, failed needle cricothyroidotomy and/or unresolved hypoxaemia occurred in 5 out of 6 patients who experienced cardiac arrest after the procedure. Whilst a direct relationship cannot be established, failed cricothyroidotomy, in combination with other physiological insults, likely contributed to patient deterioration in these cases. Our findings are consistent with other prehospital reports, where complication rates range from 8.3 to 42.0 %.^27^, ^28^, ^46^, ^47^, ^48^, ^49^, ^50^ They also underscore common procedural-complications of prehospital cricothyroidotomy. However, it is important to note that recording procedural complications was not mandatory during the study period. Therefore, complications and associated rates may be underreported.

Although cricothyroidotomy is a specialist skill for paramedics, our findings provide preliminary insights into its use and outcomes in OHCA, with potential implications for training, practice, and protocols. They also highlight knowledge gaps for future research in this area. Considering the observed survival outcomes, more research is needed to identify patient categories likely to benefit from early cricothyroidotomy. Bolstered by novel technology to measure ventilation,^51^, ^52^ such research could specifically examine associations between the timing of cricothyroidotomy and ROSC as well as other survival outcomes. Lastly, due to the rarity of prehospital cricothyroidotomy, use of standardised reporting templates and definitions, along with multi-centre research, is required to fully elucidate the value of this intervention in cardiac arrest.

### Limitations

Our study has several limitations. First, data were extracted from PCRs, which is subject to recall bias, self-reporting bias, and documentation inaccuracies. We also did not verify variables with online recordings of clinical data, such as waveform capnography, and instead relied on paramedic accounts of the procedure and success. Furthermore, as described, complications of cricothyroidotomy may have been underreported. Second, several factors, aside from technique, can impact cricothyroidotomy success. These include patient access and positioning, difficult anatomical features, and recency of training. We did not measure or control for these factors. Third, while using attempted OHCA resuscitations to calculate incidence rates aimed to address a knowledge gap, it has several limitations. These include temporal fluctuations that influence reported incidences and trends, as well as limited comparability with similar studies that have used denominators like patients undergoing intubation or advanced airway management. Finally, data for this study was obtained from a single EMS system and largely consisted of adult patients. Therefore, our findings may have limited generalisability to EMS providers with variable system characteristics and to paediatric cohorts.

## Conclusions

Over an 18-year period, this study found an increasing incidence of cricothyroidotomy in OHCA. It also identified cricothyroidotomy success to be independently associated with the presence of cardiac arrest, cricothyroidotomy technique, and patient sex. Despite high rates of ROSC and survival to hospital, few patients who underwent cricothyroidotomy in OHCA survived to hospital discharge. Given the low incidence of this intervention, future studies would benefit from consistent definitions, standardised reporting, and multi-centre research.

## Declaration of AI and AI-assisted technologies in the writing process

During the preparation of this work the author(s) used ChatGPT to check grammar and readability. After using this tool/service, the author(s) reviewed and edited the content as needed and take full responsibility for the content of the publication.

## CRediT authorship contribution statement

**Matthew Humar:** Writing – original draft, Visualization, Validation, Project administration, Methodology, Investigation, Formal analysis, Data curation, Conceptualization. **Benjamin Meadley:** Writing – review & editing, Validation, Methodology, Formal analysis, Conceptualization. **Bart Cresswell:** Writing – review & editing, Validation, Formal analysis, Conceptualization. **Emily Nehme:** Writing – review & editing. **Christopher Groombridge:** Writing – review & editing, Supervision. **David Anderson:** Writing – review & editing, Conceptualization. **Ziad Nehme:** Writing – review & editing, Visualization, Supervision, Resources, Methodology, Investigation, Formal analysis, Data curation, Conceptualization.

## Funding

None.

## Declaration of competing interest

The authors declare the following financial interests/personal relationships which may be considered as potential competing interests: Ziad Nehme reports a relationship with National Heart Foundation of Australia that includes: funding grants. Emily Nehme reports a relationship with National Health and Medical Research Council (NHMRC) Postgraduate Scholarships that includes: funding grants. The remaining authors declare that they have no known competing financial interests or personal relationships that could have appeared to influence the work reported in this paper.
